# Magnetic field quality conversion factors experimentally measured in clinical MR‐linac beams for seven MR‐compatible ionization chamber models

**DOI:** 10.1002/acm2.14613

**Published:** 2024-12-14

**Authors:** Nathan Orlando, Jennie Crosby, Carri Glide‐Hurst, Wesley Culberson, Arman Sarfehnia

**Affiliations:** ^1^ Odette Cancer Centre Sunnybrook Health Sciences Centre Toronto Ontario Canada; ^2^ Department of Radiation Oncology University of Toronto Toronto Ontario Canada; ^3^ Department of Human Oncology University of Wisconsin‐Madison Madison Wisconsin USA; ^4^ Department of Medical Physics School of Medicine and Public Health University of Wisconsin‐Madison Madison Wisconsin USA

**Keywords:** kB, magnetic field quality conversion factor, MR ionization chamber, MR‐compatible, MR‐guided radiotherapy, MR‐linac, Q, reference dosimetry

## Abstract

**Purpose:**

The purpose of this work was to experimentally quantify MR‐compatible ionization chamber response for 1.5T Elekta Unity and 0.35T ViewRay MRIdian MR‐linac systems through the determination of the magnetic field quality conversion factor, *k_B,Q_*.

**Methods:**

Seven MR‐compatible ionization chamber models from Standard Imaging and PTW were evaluated. Both the quality conversion factor *k_Q_* and the magnetic field quality conversion factor *k_B,Q_* were experimentally determined through a cross‐calibration method. Specifically, the ratio of absorbed dose measured with a reference A1SL chamber under reference conditions to corrected output measured with each test chamber at the same point of measurement allowed for the determination of *k_B,Q_*. The angular dependence of the magnetic field quality conversion factor for MR‐compatible chamber models was assessed for the 1.5T Elekta Unity system by measuring *k_B,Q_* with the chamber axis and magnetic field direction aligned at cardinal angles (0°, 90°, 180°, 270°).

**Results:**

Beam quality conversion (*k_Q_*) factors for MR‐compatible ionization chambers measured in a standard linac beam showed an average percent difference of −0.09 ± 0.18% compared to computed *k_Q_* values for their conventional chamber versions. Similarly, magnetic field quality conversion (*k_B,Q_*) factors for corresponding MR and non‐MR ionization chamber models measured using the same cross‐calibration technique demonstrated average percent differences of −0.1 ± 0.3% and 0.0 ± 0.2% for the Elekta Unity and ViewRay MRIdian, respectively. Investigation of the angular dependence of this correction factor demonstrated identical chamber response for equivalent MR‐compatible and conventional chamber models.

**Conclusions:**

This work provides critical experimental validation of MR‐compatible ionization chamber performance, with a direct comparison of measured *k_B,Q_* values to corresponding conventional chamber models demonstrating nearly equivalent chamber response. *k_B,Q_* values determined using our experimental method will serve as an important reference for upcoming MR‐linac reference dosimetry protocols and ultimately represent an important step towards accurate output calibration of MR‐linac systems.

## 1 INTRODUCTION

Magnetic resonance (MR)‐guided linear accelerator (MR‐linac) systems signify a pioneering leap in the field of radiation oncology, blending the soft tissue visualization capabilities of MR imaging with the precision of linear accelerator‐based dose delivery. This fusion offers significant advantages, including improved accuracy in targeting tumors, enhanced protection for surrounding healthy tissue, and the potential for real‐time adaptive treatment strategies. Currently, the two most widely used systems are Elekta Unity^1^ (Elekta Solutions AB, Stockholm, Sweden) and ViewRay Systems, Inc. MRIdian.^2^ The Unity MR‐linac utilizes a 1.5T magnetic field strength with a closed bore configuration while the MRIdian system uses a low‐field 0.35T magnetic field strength with a split closed bore configuration.^3^ Both systems make use of a superconducting magnet with a bore size of 70 cm and a magnetic field oriented perpendicular to the x‐ray beam direction.^3^ Emerging technologies like the MagnetTx Aurora‐RT^4^ (MagnetTx Oncology Solutions Ltd., Edmonton, AB, Canada) and the Australian MRI‐Linac program^5^ will also broaden the horizon of MR‐guided treatment platforms, although they were not included in the present work.

The presence of the strong magnetic field in MR‐linac systems results in a notable deflection in the path of electrons liberated in the patient or phantom medium by the photon beam—a phenomenon due to Lorentz forces on charged particles and one that becomes increasingly significant in lower density materials such as air.^6^ This deviation affects the trajectory of electrons within the air‐filled volumes of ionization chambers, potentially altering the charge collection dynamics crucial for accurate dose measurement.

Accurately calibrating MR‐linac systems, therefore, demands a comprehensive understanding of the magnetic field's impact on dose deposition and the development of refined dosimetry protocols that incorporate these considerations. Several initiatives have aimed to adapt existing reference dosimetry frameworks to account for magnetic field effects, leading to the formulation of protocols that extend the principles of established guidelines like the AAPM TG‐51 protocol and its addendum.^7^, ^8^ These efforts have introduced a new parameter, the magnetic field quality conversion factor, *k_B,Q_*, which adjusts for the altered chamber response in the presence of a magnetic field and is essential for maintaining dosimetric accuracy in MR‐guided radiotherapy settings.^9^, ^10^

The determination of the *k_B,Q_* factor has been the focus of extensive theoretical and computational research, employing methodologies such as Monte Carlo simulations to model the behavior of various ionization chambers under different magnetic field strengths and orientations.^9^, ^11^, ^12^, ^13^, ^14^ These studies have provided valuable insights into the expected performance of these chambers in MR‐linac environments, highlighting the influence of magnetic fields on ionization chamber response and the necessity for specific correction factors. There have also been concerted efforts to empirically validate these theoretical models and simulations through experimental studies of ionization chamber response in clinical MR‐linac beams.^15^, ^16^, ^17^, ^18^, ^19^, ^20^, ^21^

The increased prevalence of MR‐linac systems in the clinic has prompted manufacturers to develop MR‐compatible ionization chamber models specifically designed for use in MR fields. The internal structure, dimensions, and construction materials of the actual thimble remain the same between the MR‐compatible and conventional chambers of the same type. For MR‐compatible chambers manufactured by Standard Imaging, differences lie only in the triaxial cable design. Specifically, the silver‐plated, copper‐covered steel central wire used in conventional chambers is replaced with silver‐plated copper central wire in MR‐compatible versions to reduce MR imaging artifacts caused by the steel. Similarly, for the PTW T31024 MR‐compatible chamber, the standard steel sleeve material used in the conventional chamber model is replaced with aluminum to reduce artifacts during MR imaging. Due to the matching of thimble materials and dimensions between MR‐compatible and conventional chamber models, specific Monte Carlo characterization of MR‐compatible chamber response is not required. Krauss et al. experimentally determined the magnetic field quality conversion factor for four MR‐compatible chamber models (Exradin A1SLMR, A19MR, A26MR, and A28MR) in a clinical ViewRay MRIdian system via reference to absorbed dose measured with a water calorimeter.^19^ Shukla et al. characterized the same chambers via Monte Carlo simulation and experimental validation for a 6 MV linear accelerator beam and up to 1.1T external magnetic field.^22^

In this study, we present a comprehensive experimental evaluation of the performance characteristics of seven commercially available MR‐compatible ionization chambers within clinical Elekta Unity and ViewRay MRIdian MR‐linac systems. Given the lack of extensive literature for these chamber models, we first experimentally evaluated the performance of these MR‐compatible chambers against their non‐MR counterparts in terms of *k_Q_* in a standard linear accelerator in the absence of a magnetic field. We subsequently characterized the magnetic field quality conversion factors, *k_B,Q_*, for the MR‐compatible chambers, including a comparison against their non‐MR counterparts. An uncertainty budget was presented to underpin the accuracy of our findings. Overall, this comprehensive characterization of seven MR‐compatible ionization chambers represents a significant step forward in our understanding of their behavior in MR‐linac environments, facilitating the continued evolution of MR‐guided radiotherapy technology and associated dosimetry protocols.

## 2 METHODS

### 2.1 MR‐compatible ionization chambers

A total of seven MR‐compatible ionization chamber models were provided by Standard Imaging (Standard Imaging Inc., Middleton, WI, USA) and PTW (PTW Freiburg GmbH, Freiburg, Germany) for evaluation as part of this study. Table 1 lists the specific chamber models along with specifications including sensitive cavity volume and length, distance to the centroid of the sensitive volume from the tip of the chamber, and wall material and thickness. These chambers, designed specifically for use in MR fields, have identical structure, design, and dimensions compared to their conventional non‐MR counterparts. The corresponding conventional chamber model names are Exradin A1SL, A19, A12, A12S, A28, A26, and PTW T31021. The University of Wisconsin Accredited Dosimetry Calibration Laboratory (ADCL) calibrated each chamber in a reference Cobalt‐60 beam, providing chamber‐specific detector calibration coefficients, $N_{D,w}^{\mathrm{Co}60}$.

**TABLE 1:** List of MR‐compatible ionization chamber models used in this study. The chamber cavity sensitive volume, length, distance from the tip of the chamber to the centroid of the cavity, wall material, and wall thickness is provided for each chamber.

Ion chamber model	Cavity volume (cm^3^)	Cavity length (mm)	Centroid of cavity from tip (mm)	Wall material	Wall thickness (g/cm^2^)
Exradin A1SLMR	0.053	6.0	4.1	C‐552	0.211
Exradin A19MR	0.62	25.0	13.0	C‐552	0.088
Exradin A12MR	0.64	24.8	12.9	C‐552	0.088
Exradin A12SMR	0.24	10.6	5.8	C‐552	0.088
Exradin A28MR	0.125	8.3	4.5	C‐552	0.194
Exradin A26MR	0.015	2.4	2.0	C‐552	0.088
PTW T31024	0.07	4.8	3.5	PMMA	0.068
				Graphite	0.017

*Note*: The chamber cavity sensitive volume, length, distance from the tip of the chamber to the centroid of the cavity, wall material, and wall thickness is provided for each chamber.

Abbreviation: MR, magnetic resonance.

### 2.2 Experimental *k_Q_* determination

Due to the matching materials and dimensions of equivalent MR‐compatible and conventional ionization chamber models, including thimble construction, it is expected that the beam quality conversion factor, *k_Q_*, remains unchanged. To evaluate this assumption, we experimentally determined *k_Q_* for each MR‐compatible chamber model in the absence of a magnetic field using a standard 6 MV Elekta Agility (Elekta Solutions AB, Stockholm, Sweden) linear accelerator beam.

The method for experimental determination of *k_Q_* utilized a cross‐calibration approach against a reference conventional non‐MR Exradin A1SL ionization chamber. Using the standard TG‐51 formalism,^7^ the beam quality conversion factor, *k_Q_*, for each MR‐compatible test ionization chamber was calculated as the ratio of absorbed dose measured with the reference A1SL to the corrected charge reading multiplied by the detector calibration coefficient, $N_{D,w}^{\mathrm{Co}60}$, for the MR‐compatible test chamber, as shown in Equation 1,

(1) $$\;k_{Q}^{\mathrm{test}}=\frac{{\left(D_{w}^{Q}\right)}^{\mathrm{ref}\;A1\mathrm{SL}}}{{\left(MN_{D,\;w}^{\mathrm{Co}60}\right)}^{\mathrm{test}}}=\frac{{\left(Mk_{Q}N_{D,w}^{\mathrm{Co}60}\right)}^{\mathrm{ref}\;A1\mathrm{SL}}}{{\left(MN_{D,w}^{\mathrm{Co}60}\right)}^{\mathrm{test}}},$$

where *M*, the corrected charge reading is defined according to the TG‐51 addendum,^8^

(2) $$M=M_{\mathrm{raw}}\;P_{\mathrm{TP}}P_{\mathrm{ion}}P_{\mathrm{pol}}P_{\mathrm{elec}}P_{\mathrm{leak}}P_{\mathrm{rp}}.$$

Each chamber measurement was corrected for temperature and pressure ($P_{\mathrm{TP}}$), ion recombination ($P_{\mathrm{ion}}$), polarity ($P_{\mathrm{pol}}$), electrometer effects ($P_{\mathrm{elec}}$), leakage ($P_{\mathrm{leak}}$), and radial profile differences across the chamber sensitive volume length ($P_{\mathrm{rp}}$).

Utilizing a custom‐built 28 × 28 × 35 cm^3^ water tank, all measurements in the conventional linear accelerator were acquired with a 10 × 10 cm^2^ field size, defined at the machine isocenter, gantry 0°, and depth of 10 cm in water. The reference conventional A1SL chamber was placed with the centroid of the sensitive volume at the machine isocenter via in‐room laser alignment. A PTW Model T10010 UNIDOS E (PTW Freiburg GmbH, Freiburg, Germany) electrometer with standard −300 V polarization voltage was used to acquire a minimum of three charge readings, repeated until the coefficient of variation was less than 0.1%. All correction factors listed in Equation 2 were determined for each chamber following the methodologies and formula described in TG‐51 and its addendum.^7^, ^8^ The same electrometer model was used for all measurements, removing the need for an electrometer ($P_{\mathrm{elec}}$) correction factor. The beam quality conversion factor, *k_Q_*, for the reference conventional A1SL ionization chamber was calculated to be 0.9903 using the formalism described by Andreo et al.^23^ with beam quality defined by a $TPR_{10}^{20}$ of 0.678 for the 6 MV linear accelerator beam. For comparison, the *k_Q_* factor for the conventional version of each MR‐compatible chamber was also calculated using the formalism described by Andreo et al.^23^ An uncertainty budget analysis for the experimental determination of *k_Q_* in a standard linear accelerator using our described method was completed.

### 2.3 Experimental *k_B,Q_* determination

The magnetic field quality conversion factor, *k_B,Q_*, for each MR‐compatible ionization chamber model was experimentally measured in clinical 7 MV flattening filter free (FFF) 1.5 T Elekta Unity and 6 MV FFF 0.35 T ViewRay MRIdian MR‐linac systems.

Following the method described by Orlando et al.,^21^ *k_B,Q_* was determined for each MR‐compatible chamber via cross‐calibration against a reference conventional A1SL chamber, which has well‐validated Monte Carlo and experimental *k_B,Q_* data.^9^, ^18^, ^19^ Similar to the formula for *k_Q_* described in section 2.2., *k_B,Q_* was calculated as the ratio of absorbed dose measured with the conventional non‐MR A1SL chamber and the product of the corrected charge reading, *M*, the beam quality conversion factor, *k_Q_*, and the detector calibration coefficient, $N_{D,w}^{\mathrm{Co}60}$, for a given MR‐compatible chamber, as shown in Equation 3,

(3) $$k_{B,Q}^{\mathrm{test}}=\frac{{\left(D_{w}^{B,Q}\right)}^{\mathrm{ref}\;A1\mathrm{SL}}}{{\left(Mk_{Q}N_{D,w}^{\mathrm{Co}60}\right)}^{\mathrm{test}}}=\frac{{\left(Mk_{Q}k_{B,Q}N_{D,w}^{\mathrm{Co}60}\right)}^{\mathrm{ref}\;A1\mathrm{SL}}}{{\left(Mk_{Q}N_{D,w}^{\mathrm{Co}60}\right)}^{\mathrm{test}}}\;,$$

where *M* is defined as shown in Equation 2. As previously noted, the $N_{D,w}^{\mathrm{Co}60}$ factor for each chamber was provided by the University of Wisconsin ADCL. *k_B,Q_* for the reference conventional A1SL chamber was computed based on a weighted average of available Monte Carlo and experimental data, resulting in values of 0.9973 for the Elekta Unity^9^, ^18^ and 0.9985 for ViewRay MRIdian,^9^, ^19^ respectively.

The beam quality conversion factor, *k_Q_*, for MR‐compatible chambers has not been explicitly characterized via Monte Carlo simulation or experiment. Furthermore, as the static magnetic field is always present in the evaluated clinical MR‐linac systems, it was not possible to directly measure *k_Q_* for the 7 MV FFF Unity or 6 MV FFF MRIdian beams. The results of the experimental *k_Q_* determination are described in section 2.2. confirmed the assumption of matched *k_Q_* between equivalent conventional and MR‐compatible chamber models with equal dimensions. As such, *k_Q_* values for conventional ionization chamber models computed using the formalism described by Andreo et al.^23^ were directly applied to the corresponding MR‐compatible chamber models. These calculations utilized a beam quality definition via $TPR_{10}^{20}$ of 0.699 and 0.643 for the Elekta Unity and ViewRay MRIdian MR‐linac systems, respectively, as measured by Orlando *et al*.^21^

**FIGURE 1:**
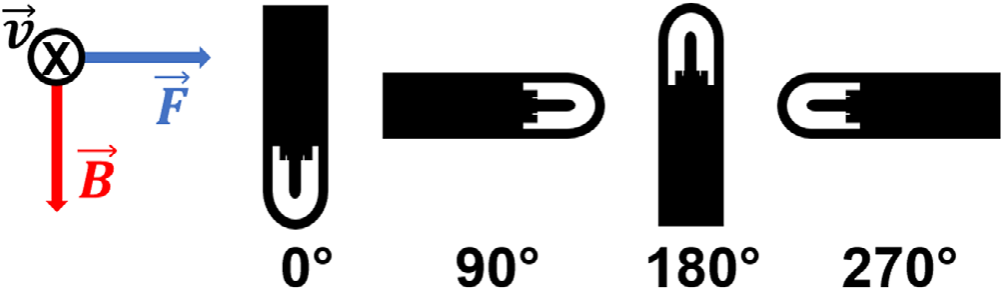
Diagram of the Exradin A1SL chamber model demonstrating ionization chamber angular orientation (0°, 90°, 180°, and 270°) with respect to the static magnetic field ($\vec{B}$), x‐ray beam ($\vec{v}$), and Lorentz force directions ($\vec{F}$).

All measurements utilized a 10 × 10 cm^2^ field, defined at the machine isocenter, gantry angle of 0°, and depth of 10 cm in water. Ionization chambers were aligned such that the centroid of the sensitive collecting volume was placed at the machine isocenter. Chamber angular orientation relative to the static magnetic field has been shown to have a large influence on charge collection and thus *k_B,Q_*.^9^, ^18^ To minimize this effect, the default orientation placed the ionization chamber axis parallel to the static magnetic field direction (so‐called 0° orientation) and perpendicular to the radiation beam, as shown in Figure 1. Measurements were first obtained with the conventional reference A1SL chamber, followed by measurements with each MR‐compatible chamber at the same point of measurement. A PTW Model T10010 UNIDOS E electrometer with standard ‐300 V polarization voltage was used for all measurements. A minimum of three charge readings were acquired for each chamber model, repeated as required until the coefficient of variation was less than 0.1%. All correction factors listed in Equation 2 were determined for each chamber following the methodologies and formula described in TG‐51 and its addendum.^7^, ^8^ Reference A1SL output measurements were repeated at the start and end of each measurement session to assess the output stability of each MR‐linac system for uncertainty budget analysis.

Measurements with the Elekta Unity system utilized a custom‐built 28 × 28 × 35 cm^3^ MR‐compatible water tank. The tank has been described in detail in our earlier publications.^18^, ^21^ Custom chamber holders were fixed to a stage on the water tank, allowing vertical movement of the ionization chambers. Chamber alignment to the machine isocenter for the Elekta Unity was accomplished using the system's calibrated onboard MV electronic portal imaging device (EPID). EPID images of each chamber model were acquired at a gantry angle of 0° (superior/inferior, left/right, and rotational alignment) and 90° (vertical alignment) to ensure accurate positioning of the chamber sensitive volume at the machine isocentre for each measurement.

ViewRay MRIdian measurements utilized a commercial 30 × 40 × 38 cm^3^ CNMC WP‐3040 water tank (CNMC Company Inc., Nashville, TN, USA), including a universal chamber holder. Alignment of the ionization chamber point of measurement to the machine isocenter was accomplished via calibrated lasers outside of the MR‐linac bore. After alignment of the chamber to the laser isocenter, a translation of 155 cm into the bore moved the chamber point of measurement to the machine isocenter position.

An uncertainty budget analysis for the experimental determination of the magnetic field quality conversion factor, *k_B,Q_*, in Unity and MRIdian MR‐linac systems using our described method was completed.

### 2.4 Angular dependence of $k_{B,Q}$

To assess the sensitivity of response of an MR‐compatible ionization chamber as a function of its orientation relative to the static magnetic field direction and to compare this against its conventional counterpart, the angular dependence of *k_B,Q_* was evaluated as described in Orlando et al.^21^ Utilizing the cross‐calibration method shown by Equation 3, the magnetic field quality conversion factor, *k_B,Q_*, was measured for each MR‐compatible chamber with the chamber axis positioned antiparallel (180°) as well as perpendicular (90° & 270°) to the static magnetic field direction, as shown in Figure 1. In each case, the reference absorbed dose was measured with the conventional A1SL ionization chamber with the chamber axis positioned in the standard parallel (0°) orientation relative to the magnetic field direction. A custom cylindrical insert for the Elekta Unity water tank^18^ allowed for rotation of the ionization chamber about the machine isocenter, thereby allowing for variation of the chamber direction relative to the static magnetic field. While the water tank featured etchings to denote the chamber angle of rotation, MV EPID imaging was used to confirm the final rotational alignment for each measurement.

The magnitude of change in ionization chamber response as the chamber orientation varies with the magnetic field direction has been shown to be higher for the high‐field 1.5 T Elekta Unity system compared to the 0.35 T ViewRay MRIdian.^9^, ^24^, ^25^ As such, the characterization of *k_B,Q_* as a function of chamber orientation versus the magnetic field was completed for only the Elekta Unity.

## 3 RESULTS

### 3.1 Beam quality conversion factor, $k_{Q}$

A comprehensive uncertainty budget analysis for our cross‐calibration beam quality conversion factor measurement method was completed, as shown in Table 2. The overall uncertainty for these *k_Q_* measurements completed in a standard 6 MV linear accelerator was 0.75% (k = 1). This overall uncertainty was dominated by the 0.6% uncertainty in the reference A1SL *k_Q_* factor as provided by Andreo et al.^23^ The use of a cross‐calibration approach allowed for the reduction of uncertainty due to $N_{D,w}^{\mathrm{Co}60}$ factors by considering the ratio of these values for the reference A1SL and test MR‐compatible chamber. As the ADCL standard ionization chamber used was the same for all calibrations, it is associated uncertainty could be removed, reducing the total uncertainty of ${\left(N_{D,w}^{\mathrm{Co}60}\right)}^{\mathrm{ref}\;A1\mathrm{SL}}/{\left(N_{D,w}^{\mathrm{Co}60}\right)}^{\mathrm{test}}$ to 0.25%, as described in Orlando et al.^21^ Other potential uncertainty reductions due to correlated components were not considered, making this a conservative estimate of overall uncertainty. The use of a single electrometer model for all measurements removed the need for $P_{\mathrm{elec}}$ correction and its associated uncertainty. If uncertainties due to the A1SL *k_Q_* and $N_{D,w}^{\mathrm{Co}60}$ ratio were disregarded, the remaining user‐dependent experimental uncertainty was only 0.36%.

**TABLE 2:** Uncertainty budget (*k* = 1) for *k*_Q_ determination for MR‐compatible ionization chambers via cross calibration to a reference conventional A1SL chamber in a standard 6 MV Elekta Agility linear accelerator beam.

Component of uncertainty	%
**General components**	
$\;{\left(N_{D,w}^{\mathrm{Co}60}\right)}^{\mathrm{ref}\;A1\mathrm{SL}}/{\left(N_{D,w}^{\mathrm{Co}60}\right)}^{\mathrm{test}}$	0.26%
**Reference A1SL chamber**	
Standard error of the mean	0.05%
Depth setting	0.15%
Alignment of chamber axis	0.10%
$k_{Q}$	0.60%
$P_{\mathrm{pol}}$	0.05%
$P_{\mathrm{ion}}$	0.10%
$P_{\mathrm{TP}}$	0.05%
$P_{\mathrm{leak}}$	0.05%
$P_{\mathrm{rp}}$	0.05%
**MR‐compatible test chamber**	
Standard error of the mean	0.15%
Depth setting	0.15%
Alignment of chamber axis	0.10%
$P_{\mathrm{pol}}$	0.05%
$P_{\mathrm{ion}}$	0.10%
$P_{\mathrm{TP}}$	0.05%
$P_{\mathrm{leak}}$	0.05%
$P_{\mathrm{rp}}$	0.05%
**COMBINED (*k* = 1)**	**0.75%**

Abbreviation: MR, magnetic resonance.

The beam quality conversion factor, *k_Q_*, for seven MR‐compatible ionization chamber models determined through a cross‐calibration method against a reference conventional A1SL chamber is provided in Table 3. For reference, quality conversion factors computed using the method described by Andreo et al.^23^ for corresponding conventional ionization chamber models are provided. All *k_Q_* measurements were completed using a standard 6 MV Elekta Agility linear accelerator.

**TABLE 3:** Quality conversion factor, *k_Q_*, values for seven MR‐compatible ionization chamber models, experimentally measured in a standard 6 MV linear accelerator beam. Calculated *k_Q_* values for the corresponding conventional chamber models are provided for comparison. Percent difference from the established *k_Q_* value is provided for each chamber.

Ion chamber model	Measured *k_Q_*	Andreo et al. *k_Q_* ^a^	Percent difference (%)
Exradin A1SLMR	0.992	0.9903	0.17%
Exradin A19MR	0.991	0.9906	0.04%
Exradin A12MR	0.992	0.9918	0.02%
Exradin A12SMR	0.989	0.9916	−0.26%
Exradin A28MR	0.991	0.9917	−0.07%
Exradin A26MR	0.989	0.9921	−0.31%
PTW T31024	0.987	0.9889	−0.19%

*Note*:^a^Calculated *k*_Q_ values for the equivalent conventional chambers were calculated using the formalism described in Andreo et al. (2020) with a $TPR_{10}^{20}$ value of 0.678.

Abbreviation: MR, magnetic resonance.

### 3.2 Magnetic field quality conversion factor, $k_{B,Q}$

Experimental determination of the magnetic field quality conversion factors, *k_B,Q_*, for MR‐compatible ionization chamber models utilized the same cross‐calibration method as described in Orlando et al.^21^ As a result, experimental uncertainty in *k_B,Q_* determination was the same, with combined (k = 1) uncertainty of 0.71% and 0.72% for measurements in the Elekta Unity and ViewRay MRIdian MR‐linac systems, respectively.^21^ Readers are directed to Orlando et al. to view the comprehensive uncertainty budget analysis table.^21^

Table 4 lists the magnetic field quality conversion, *k_B,Q_*, factors measured experimentally in clinical Elekta Unity and ViewRay MRIdian MR‐linac systems for seven MR‐compatible ionization chamber models. These values correspond to the parallel (0°) orientation where the chamber's long axis is in line with the magnetic field direction.

**TABLE 4:** Magnetic field quality conversion factor, *k_B,Q_*, values for seven MR‐compatible ionization chamber models. *k_B,Q_* factors were experimentally determined using clinical ViewRay MRIdian and Elekta Unity MR‐linac systems with chambers oriented parallel to the magnetic field direction (0° orientation).

	*k_B,Q_*	
Ion Chamber Model	Elekta Unity^a^	ViewRay MRIdian^b^
Exradin A1SLMR	0.997	1.003
Exradin A19MR	1.003	0.998
Exradin A12MR	1.002	1.001
Exradin A12SMR	0.994	0.998
Exradin A28MR	1.000	0.998
Exradin A26MR	0.997	0.996
PTW T31024	0.989	0.997

^a^ A combined uncertainty of 0.71% (k = 1) applies to all *k_B,Q_* data for Elekta Unity.

^b^ A combined uncertainty of 0.72% (k = 1) applies to all *k_B,Q_* data for ViewRay MRIdian.

Abbreviations: MR, magnetic resonance; MR‐linac, magnetic resonance guided linear accelerator

**FIGURE 2:**
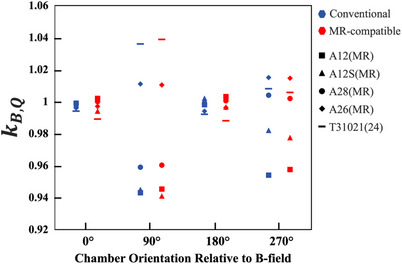
Scatter plot of the magnetic field quality conversion factor, *k_B,Q_*, for corresponding MR‐compatible and conventional^21^ ionization chamber pairs (*n* = 5) as chamber orientation varied with respect to the static magnetic field direction. Experimental uncertainty for each measured value was 0.71%. MR, magnetic resonance.

Magnetic field quality conversion factors measured in a high‐field 1.5 T Elekta Unity MR‐linac as the chamber long axis varies between 0°, 90°, 180°, and 270° orientations relative to the static magnetic field for seven MR‐compatible ionization chamber models are provided in Table 5. To investigate the differences in angular dependence a scatter plot of *k_B,Q_* for corresponding MR‐compatible (this study) and conventional (Orlando et al.^21^) ionization chamber model pairs is shown in Figure 2.

**TABLE 5:** Variation of the magnetic field quality conversion factor, *k_B,Q_*, for MR‐compatible ionization chamber models measured in the 1.5 T Elekta Unity system as chambers were oriented 0° (parallel), 90°, 180° (anti‐parallel), and 270° relative to the static magnetic field direction. The percent deviation in *k_B,Q_* with respect to the standard 0° orientation are shown in brackets.

	*k_B,Q_*			
Ion Chamber Model	0°	90°	180°	270°
Exradin A1SLMR	0.997	0.971 (−2.6%)	0.996 (−0.1%)	0.996 (−0.1%)
Exradin A19MR	1.003	0.951 (−5.2%)	1.002 (−0.1%)	0.956 (−4.7%)
Exradin A12MR	1.002	0.945 (−5.7%)	1.003 (0.1%)	0.958 (−4.4%)
Exradin A12SMR	0.994	0.941 (−5.3%)	0.997 (0.3%)	0.977 (−1.7%)
Exradin A28MR	1.000	0.960 (−4.0%)	1.001 (0.1%)	1.002 (0.2%)
Exradin A26MR	0.997	1.010 (1.3%)	0.996 (−0.1%)	1.015 (1.8%)
PTW T31024	0.989	1.039 (5.1%)	0.988 (−0.1%)	1.006 (1.7%)

Abbreviation: MR, magnetic resonance.

## 4 DISCUSSION

### 4.1 Beam quality conversion factor, $k_{Q}$

The quality conversion factor, *k_Q_*, values determined for the seven MR‐compatible ionization chambers via our experimental method and the equivalent conventional ionization chambers calculated using the method described by Andreo et al.^23^ agreed closely, to well within the uncertainty of the measurements for all chamber models. The maximum percent error was –0.31%, with the average deviation being (–0.09 ± 0.18)%. This close agreement confirms the assumption that due to identical chamber dimensions, as well as thimble design and construction materials between the MR‐compatible and conventional models, chamber response in a standard linear accelerator beam may be considered equivalent, within experimental uncertainty.

Determination of these *k_Q_* values was done using a standard 6 MV Elekta Agility linear accelerator beam. As the magnetic field is always present in the clinical MR‐linac systems used in our study, we could not directly measure the *k_Q_* values for the 7 MV FFF Elekta Unity or 6 MV FFF ViewRay MRIdian beams. Although *k_Q_* is required for our experimental magnetic field quality conversion factor determination (Equation 3), demonstration of the equivalence of the MR‐compatible and conventional ionization chamber response in a standard linear accelerator beam (close in beam quality to that of the MR‐linac beams) justified the use of conventional chamber model *k_Q_* values calculated via the Andreo method,^23^ thus alleviating the need for direct *k_Q_* measurement in the MR‐linac.

### 4.2 Magnetic field quality conversion factor, $k_{B,Q}$

The magnetic field quality conversion factor, *k_B,Q_*, values for MR‐compatible ionization chamber models were first compared to the equivalent conventional chamber models, as shown in Table 6. Orlando *et al*. experimentally determined *k_B,Q_* values for the equivalent conventional versions of five of the seven MR chambers using the same experimental method as described in this manuscript for both the Elekta Unity and ViewRay MRIdian systems.^21^ *k_B,Q_* values for all MR/non‐MR ionization chamber pairs agreed to within uncertainty. For the Elekta Unity MR‐linac, the maximum percent error was −0.5%, with an average of (−0.1 ± 0.3)%, while the maximum error was 0.3% with an average of (0.0 ± 0.2)% for the ViewRay MRIdian, indicating identical chamber response in the MR‐field, to within experimental uncertainty. These observed differences could be explained in part due to small variations in chamber construction within the build tolerances for otherwise identical thimble materials and dimensions. As shown by Woodings et al.,^26^ the standard deviation in measured magnetic field correction factors for 12 PTW 30013 and 13 FC65‐G chambers oriented parallel to the magnetic field was 0.19% and 0.15%, respectively, similar in magnitude to the measured standard deviations in *k_B,Q_* between conventional and MR‐compatible chambers in this study. Iakovenko et al. experimentally measured *k_B,Q_* for conventional Exradin A1SL and A19 ionization chamber models using a similar cross‐calibration approach for only the Elekta Unity MR‐linac,^18^ demonstrating 0.4% and 0.8% percent error relative to our measured values for the A1SLMR and A19MR. Utilizing alanine electron paramagnetic resonance dosimetry, Pojtinger et al. experimentally measured *k_B,Q_* for the PTW T31021 ionization chamber in the parallel orientation.^20^ The published value corresponded to a percent error of only 0.1% compared to our experimentally derived *k_B,Q_* for the equivalent PTW T31024 MR‐compatible chamber model, demonstrating excellent agreement to well‐within uncertainty.

**TABLE 6:** Our experimentally determined magnetic field quality conversion factor, *k_B,Q_*, values compared to published Monte Carlo and experimental results.

Ion chamber model	Source	Method	*k_B,Q_* (1.5 T)	*k_B,Q_* (0.35 T)
Exradin A1SLMR	Present Study	Experiment	0.997	1.003
	^a^Malkov & Rogers^9^	Monte Carlo	0.9966 (−)	0.9992 (0.4%)
	^a^Margaroni et al.^13^	Monte Carlo	1.0026 (−0.6%)	–
	^b^Khan et al.^14^	Monte Carlo	–	0.999 (0.4%)
	^a^Iakovenko et al.^18^	Experiment	0.993 (0.4%)	–
	^b^Krauss et al.^19^	Experiment	–	0.9975 (0.6%)
Exradin A19MR	Present Study	Experiment	1.003	0.998
	^a^Malkov & Rogers^9^	Monte Carlo	1.0007 (0.2%)	1.0006 (−0.3%)
	^a^O'Brien et al.^11^	Monte Carlo	1.005 (−0.2%)	–
	^a^Iakovenko et al.^18^	Experiment	0.995 (0.8%)	–
	^b^Krauss et al.^19^	Experiment	–	0.9976 (−)
Exradin A12MR	Present Study	Experiment	1.002	1.001
	^a^Orlando et al.^21^	Experiment	0.999 (0.3%)	1.001 (−)
	^a^Malkov & Rogers^9^	Monte Carlo	0.9983 (0.4%)	1.0001 (0.1%)
	^a^Margaroni et al.^13^	Monte Carlo	1.0060 (−0.4%)	–
	^b^Khan et al.^14^	Monte Carlo	–	0.989 (1.2%)
Exradin A12SMR	Present Study	Experiment	0.994	0.998
	^a^Orlando et al.^21^	Experiment	0.998 (−0.4%)	1.000 (−0.2%)
	^a^Malkov & Rogers^9^	Monte Carlo	0.9984 (−0.4%)	0.9999 (−0.2%)
Exradin A28MR	Present Study	Experiment	1.000	0.998
	^a^Orlando et al.^21^	Experiment	0.999 (0.1%)	0.995 (0.3%)
	^b^Khan et al.^14^	Monte Carlo	–	0.992 (0.6%)
	^b^Krauss et al.^19^	Experiment	–	0.9962 (0.2%)
Exradin A26MR	Present Study	Experiment	0.997	0.996
	^a^Orlando et al.^21^	Experiment	0.996 (0.1%)	0.996 (−)
	^a^Margaroni et al.^13^	Monte Carlo	1.0034 (−0.6%)	–
	^b^Khan et al.^14^	Monte Carlo	–	0.991 (0.5%)
	^b^Krauss et al.^19^	Experiment	–	0.9939 (0.2%)
PTW T31024	Present Study	Experiment	0.989	0.997
	^a^Orlando et al.^21^	Experiment	0.994 (−0.5%)	0.998 (−0.1%)
	^a^Cervantes et al.^12^	Monte Carlo	1.016 (−2.7%)	–
	^a^Margaroni et al.^13^	Monte Carlo	1.0019 (−1.3%)	–
	^a^Pojtinger et al.^20^	Experiment	0.9885 (0.1%)	–
	^a^Krauss et al.^19^	Experiment	–	0.9937 (0.3%)

*Note*: ^a^ *k_B,Q_* was determined for the equivalent non‐MR conventional chamber model.

^b^ *k_B,Q_* was determined for the MR‐compatible chamber model. The percent difference is computed for each comparison with the literature value serving as the reference. All comparisons are for chambers positioned parallel to the magnetic field (0° orientation).

Abbreviation: MR, magnetic resonance.

As shown in Table 6, there were several studies utilizing Monte Carlo simulation to estimate *k_B,Q_* for conventional versions of the chambers evaluated in this study. Malkov & Rogers simulated the 1.5 T Elekta Unity system and the Cobalt‐60 version of the 0.35 T ViewRay MRIdian system to investigate the response of several conventional chamber models, including the Exradin A1SL, A19, A12, and A12S.^9^ Compared to our experimental *k_B,Q_* data in the 0° parallel orientation for MR‐compatible versions of these chambers, we calculated average percent errors of 0.1 ± 0.3% and 0.0 ± 0.3% for Elekta Unity and ViewRay MRIdian, respectively, with values for all chamber models agreeing to within uncertainty. To further contextualize these results, it is important to note that the magnetic field quality conversion factor calculated by Malkov & Rogers for the A1SL chamber was one of two sources of data for the weighted average *k_B,Q_* of our reference A1SL. This connection may contribute to the excellent agreement we observed. Similar simulations of the Elekta Unity MR‐linac were completed by Margaroni et al. to calculate *k_B,Q_* for a number of conventional ionization chamber models, including the Exradin A1SL, A12, A26, and PTW T31021.^13^ Comparing those results against our experimentally measured *k_B,Q_* values for the MR‐compatible chambers shows good agreement with an average percent difference of (‐0.7 ± 0.4)% for the parallel orientation.

Interestingly, an agreement between our experimental *k_B,Q_* results for the PTW T31024 ionization chamber and two separate Monte Carlo simulations of the corresponding PTW T31021 chamber did not agree within uncertainty.^12^, ^13^ However, our results did agree to within 0.5% with three separate experimental characterizations of the PTW T31021 chamber using different measurement techniques, as shown in Table 6.^19^, ^20^, ^21^ Furthermore, the Monte Carlo‐simulated *k_B,Q_* values calculated by Margaroni et al.^13^ and Cervantes et al.^12^ were higher in magnitude compared to other Monte Carlo studies and experiments across all chamber models investigated. Along with the 1.4% difference between these two separate Monte Carlo calculations, this suggests an inconsistency in the simulation data compared to chamber performance in clinical MR‐linac beams.

To our knowledge, there are very few studies characterizing the response of these MR chamber models in clinical MR‐linac systems. Krauss et al. experimentally measured the magnetic field quality conversion factor in the 0.35 T ViewRay MRIdian MR‐linac for several cylindrical MR‐compatible ionization chamber models via comparison to water calorimetry as a primary standard.^19^ Overlap with chambers characterized in our study included the Exradin A1SLMR, A19MR, A28MR, and A26MR. The average percent difference between our experimental *k_B,Q_* values for these four MR‐compatible chamber models was (0.25 ± 0.25)%, with all values agreeing to within uncertainty. The reduced experimental uncertainty afforded by the direct comparison to water calorimetry measurement for the method described by Krauss *et al*. and the corresponding close agreement to our own values serve to further validate our cross‐calibration technique. Khan et al. calculated beam quality correction factors for four MR‐compatible cylindrical ionization chamber models (Exradin A1SLMR, A12MR, A26MR, and A28MR) in the 0.35T ViewRay MRIdian system via Monte Carlo simulation.^14^ While Khan et al. report values for $k_{Q_{msr}}^{B,f_{msr}}$, *k_B,Q_* as reported in this study they could be computed by dividing the reported $k_{Q_{msr}}^{B,f_{msr}}$ factor by the calculated *k_Q_* factor for the respective chamber, assuming $TPR_{10}^{20}$ of 0.643 for the ViewRay MRIdian system. In the 0° orientation, the average percent difference in *k_B,Q_* between the simulation results and our experimental results was (0.68 ± 0.36)%, with all chamber models except the A12MR agreeing to within uncertainty. Shukla et al. investigated the response of the same Exradin A1SLMR, A19MR, A26MR, and A28MR ionization chamber models through both Monte Carlo simulation and experiment.^22^ Their study utilized a conventional 6 MV linear accelerator with an external electromagnet allowing for up to only 1.1T field strength, making direct comparisons not possible.

The angular dependence of the magnetic field quality conversion factor for MR‐compatible ionization chamber models was evaluated for the 1.5 T Elekta Unity MR‐linac as described in section 2.4. Table 5 highlights this dependence as the orientation of the long axis of the ionization chamber varies with the magnetic field direction, confirming previously reported results that the *k_B,Q_* factor is minimized with the chamber axis parallel or anti‐parallel to the magnetic field,^9^, ^18^, ^21^ and that these two parallel orientations are equivalent.^21^ Critically, the angular dependence of *k_B,Q_* for equivalent MR‐compatible and conventional ionization chamber pairs agrees closely. Using the same experimental technique, including the same custom‐made water tank, Orlando *et al*. assessed the angular dependence of eleven conventional ionization chamber models, including five that overlap with the MR‐compatible chambers evaluated in this study (Exradin A12, A12S, A26, A28, and PTW T31021).^21^ The average percent difference in *k_B,Q_* between corresponding MR‐compatible and conventional ionization chambers was (−0.1 ± 0.4)%, (0.0 ± 0.3)%, (0.0 ± 0.4)%, and (−0.1 ± 0.3)% for 0°, 90°, 180°, and 270° orientations, respectively. Regardless of the orientation, all corresponding values agreed within uncertainty. Thus, although the magnetic field has the largest impact on charge collection when oriented perpendicular to the ionization chamber axis, and this impact can result in increased or decreased charge collection depending on the chamber design, the chamber response is remarkably similar for given MR‐compatible and conventional chamber pairs. As noted, these measurements were performed solely in a 1.5T Elekta Unity system due to resource limitations. It is expected that the lower field strength MRIdian system (0.35T) further reduces the magnitude of angular dependence of chamber response.^9^, ^24^, ^25^

As the same experimental procedure was used, limitations described in Orlando et al. also apply to the present study.^21^ This includes the inability to explicitly assess how chamber‐to‐chamber variations resulting from construction differences impact the magnetic field quality conversion factor, which has been shown to be on the order of 0.2% for conventional ionization chamber models.^26^ Given a clear lack of characterization data for MR‐compatible chamber response, the comparisons outlined in this study serve to further validate simulation results while also showing for the first time, to our knowledge, a direct comparison between corresponding MR‐compatible and conventional ionization chamber models. The generally excellent agreement between *k_B,Q_* values for MR‐compatible chamber models and conventional chamber models determined via Monte Carlo simulation or experimentation in the literature highlights the clinical equivalence of chamber response in the Unity and MRIdian MR‐linac beams evaluated. Furthermore, there was minimal difference in the angular dependence of ionization chamber response with the MR‐compatible chamber models in comparison to equivalent conventional chambers for the 1.5T MR‐linac. As highlighted in this manuscript, the conventional and MR‐compatible chambers have equivalent responses, and thus both chamber types are equally valid for dosimetry in the MR‐linac environment. The main benefit of MR‐compatible chambers is the reduced artifacts produced during MR imaging of the chambers. In addition, while conventional chamber models can be utilized in the MR environment following rigorous MR screening, MR‐compatible chambers have been designed with MR fields in mind, providing peace of mind for use in the MR‐linac environment. Examining chamber performance in other MR‐linac systems would be advantageous.

## 5 CONCLUSION

Seven MR‐compatible ionization chamber models were experimentally characterized in clinical Elekta Unity and ViewRay MRIdian MR‐linac systems via determination of the magnetic field quality conversion factor, *k_B,Q_*. Compared to *k_B,Q_* values for corresponding conventional ionization chamber models in the literature, determined via Monte Carlo and experimental methods, our values agreed to within uncertainty for nearly all cases. This study provides a critical characterization of MR‐compatible ionization chamber performance in the two most used MR‐linac systems where data is limited. The provided *k_B,Q_* are important for accurate output calibration of MR‐guided radiotherapy systems and will be a key source of data for upcoming MR‐linac reference dosimetry protocols.

## AUTHOR CONTRIBUTIONS

Nathan Orlando contributed scientific design, data acquisition, data analysis; and drafted the manuscript. Jennie Crosby and Carri Glide‐Hurst contributed scientific design, data acquisition, and manuscript review. Wesley Culberson coordinated ionization chamber calibration and completed the manuscript review. Arman Sarfehnia contributed scientific design, manuscript drafting, and manuscript review.

## CONFLICT OF INTEREST STATEMENT

Standard Imaging and PTW provided funding to cover the cost of shipping the loaned MR‐compatible ionization chambers as well as the cost of calibration at the University of Wisconsin ADCL. No financial compensation was paid to the authors, and the vendors had no input on the experimental procedure or content of this manuscript. Dr. Glide‐Hurst discloses research collaborations with Modus Medical Devices, Leo Cancer Care, Inc., Raysearch Laboratories, and GE Healthcare.
